# Novel Biomarkers of Dynamic Blood PD-L1 Expression for Immune Checkpoint Inhibitors in Advanced Non-Small-Cell Lung Cancer Patients

**DOI:** 10.3389/fimmu.2021.665133

**Published:** 2021-04-16

**Authors:** Qiao Yang, Mingjing Chen, Jiaoyang Gu, Kai Niu, Xianlan Zhao, Linpeng Zheng, Zihan Xu, Yongxin Yu, Feng Li, Lingxin Meng, Zhengtang Chen, Wenlei Zhuo, Luping Zhang, Jianguo Sun

**Affiliations:** ^1^ Cancer Institute, Xinqiao Hospital, Army Medical University, Chongqing, China; ^2^ Department of Ultrasound, The 941st Hospital of the PLA Joint Logistic Support Force, Xining, China; ^3^ Department of Oncology, Liangping People’s Hospital, Liangping, China

**Keywords:** blood PD-L1, immune checkpoint inhibitors, NSCLC, exosome, biomarker

## Abstract

**Background:**

Immune checkpoint inhibitors (ICIs) have become a high-profile regimen for malignancy recently. However, only a small subpopulation obtains long-term clinical benefit. How to select optimal patients by reasonable biomarkers remains a hot topic.

**Methods:**

Paired tissue samples and blood samples from 51 patients with advanced malignancies were collected for correlation analysis. Dynamic changes in blood PD-L1 (bPD-L1) expression, including PD-L1 mRNA, exosomal PD-L1 (exoPD-L1) protein and soluble PD-L1 (sPD-L1), were detected after 2 months of ICIs treatment in advanced non-small-cell lung cancer (NSCLC) patients. The best cutoff values for progression-free survival (PFS) and overall survival (OS) of all three biomarkers were calculated with R software.

**Results:**

In 51 cases of various malignancies, those with positive tissue PD-L1 (tPD-L1) had significantly higher PD-L1 mRNA than those with negative tPD-L1. In 40 advanced NSCLC patients, those with a fold change of PD-L1 mRNA ≥ 2.04 had better PFS, OS and best objective response (bOR) rate. In addition, a fold change of exoPD-L1 ≥ 1.86 was also found to be associated with better efficacy and OS in a cohort of 21 advanced NSCLC cases. The dynamic change of sPD-L1 was not associated with efficacy and OS. Furthermore, the combination of PD-L1 mRNA and exoPD-L1 could screen better patients for potential benefit from ICIs treatment.

**Conclusion:**

There was a positive correlation between bPD-L1 and tPD-L1 expression. Increased expression of PD-L1 mRNA, exoPD-L1, or both in early stage of ICIs treatment could serve as positive biomarkers of efficacy and OS in advanced NSCLC patients.

## Introduction

Immune checkpoint inhibitors (ICIs) treatment has become an increasingly high-profile regimen for malignancies since 2013. Patients with malignancies obtain remarkable survival benefits from ICIs treatment, for example, when antibodies against programmed cell death 1 (PD-1) and programmed cell death ligand 1 (PD-L1) are compared to traditional chemotherapy in non-small-cell lung cancer (NSCLC) (1, 2). As effective as ICIs treatment can be, only 10–40% of patients obtain dramatic responses (3), and the five-year overall survival (OS) rate of ICIs treatment ranges from 15.5% to 41% in advanced malignancies (4–6). Using single or multiple biomarkers to select patients who could benefit from ICIs was the focus in the current study.

To date, various biomarkers, including tumor tissue PD-L1 (tPD-L1) expression, tumor mutation burden (TMB), tumor neoantigen burden (TNB), high microsatellite instability (MSI-high), deficient mismatch repair (dMMR), tumor-infiltrating lymphocytes (TIL), T-cell receptor clonality, effector T-cell gene signature, DNA damage and repair genes (DDR), intestinal microbiota, etc. have been demonstrated to be associated with a better response rate and prolonged survival (7–10).

In the tumor microenvironment (TME), the PD-L1 protein is expressed on the surface of tumor cells (TCs) or immune cells (ICs). Its binding to PD-1 leads to the impairment of the antitumor function of T cells, similar to a blockade in the flow of a pipeline. Anti-PD-1/anti-PD-L1 therapy could move the blockade away and restore the flow (11). Hence, the detection of pretreatment PD-L1 protein expression on TCs or ICs by immunohistochemistry (IHC) is the most frequently used predictive biomarker in clinical practice. Previous studies KEYNOTE 024 and IMpower 110 have demonstrated that NSCLC patients with higher tPD-L1 expression could obtain better clinical benefits, including objective response rate (ORR), progression-free survival (PFS) and OS (12, 13). In addition, the dynamic changes in tPD-L1 expression help distinguish responders from non-responders (14, 15). However, in the CHECKMATE-026 study (16), the nivolumab subgroup did not have a PFS benefit compared with the platinum-based chemotherapy subgroup in patients with 5% or higher tPD-L1 expression. Hence, tPD-L1 expression is a controversial predictive biomarker in the clinic. There are several reasons. First, there is heterogeneity of PD-L1 protein expression in the TME. The PD-L1 protein in the TME includes constitutive expression from the activation of some oncogenic pathways or chromosomal abnormalities (17, 18), and inducible expression by the activation of NF-κB or IFN-γ secreted by infiltrating lymphocytes (19, 20). Second, previous treatment had an effect on tPD-L1 expression. A study demonstrated that radiotherapy upregulated tPD-L1 expression (21), while EGFR-TKIs downregulated tPD-L1 expression (22). Third, there is no standard measure of tPD-L1 expression, for the inconsistency and subjectivity between different detection kits. In conclusion, tPD-L1 expression may not be a robust predictive biomarker.

Liquid biopsy is an emerging assay to obtain tumor-related molecular information. The sample sources of liquid biopsy included cerebrospinal fluid, saliva, pleural effusion, blood, ascites, urine, etc. Compared to tissue biopsy, liquid biopsy is noninvasive and convenient, which could help obtain multiple biopsies to monitor the molecular changes during ICIs treatment. In addition, liquid biopsy could help to reduce the effect of tumor heterogeneity. Some blood biomarkers, such as blood TMB (bTMB) (23), derived neutrophil/(leukocyte minus neutrophil) ratio (24), circulating exosomal PD-L1 (exoPD-L1) protein expression (25), soluble PD-L1 (sPD-L1) (26) have been explored to predict efficacy of ICIs treatment. However, these studies showed controversial results in different research centers.

To explore the value of bPD-L1 in ICIs treatment, the current study was designed to detect multi-modal bPD-L1 expression (including PD-L1 mRNA, exoPD-L1 and sPD-L1), evaluate the correlation between tPD-L1 and bPD-L1, and monitor the dynamic changes in early stage of ICIs treatment.

## Materials and Methods

### Study Design and Patients

Paired tumor tissue samples and blood samples, as well as clinicopathologic features were obtained from 51 various malignant tumor patients (ClinicalTrials.gov, NCT02890849). Repeated blood biopsies from forty other advanced NSCLC patient with anti-PD-1/anti-PD-L1 therapy were collected at baseline and at two months after the first intravenous transfusion (ClinicalTrials.gov, NCT03073902). In addition, blood samples from ten healthy donors (HDs) were collected. All patients and HDs provided informed consent. All tissue samples underwent overnight fixation in 10% phosphate-buffed formalin and then were processed and embedded in paraffin blocks for further analysis. All blood samples were centrifuged for 10 minutes at 2000 × g to obtain plasma and then stored at -80°C for further analysis. This study was approved by the ethics committee of the Xinqiao Hospital of Army Medical University (2016-No.054-01, 2017-No.011-01). The best objective response (bOR) to anti-PD-1/anti-PD-L1 antibody treatment was determined by iRECIST (27) and included complete response (CR), partial response (PR), stable disease (SD) and progressive disease (PD). PFS was defined as the time from the first dose of ICIs treatment to PD. OS was defined as the time from the first dose of ICIs treatment to death for any reason.

### PD-L1 IHC Staining and Scoring

PD-L1 IHC staining was conducted on 3 μm thick sections of formalin-fixed paraffin embedded (FFPE) tumor blocks according to the VENTANA SP142 PD-L1 immunohistochemistry assay (Ventana, AZ, USA). The score of tPD-L1 expression on both TCs and tumor-infiltrating ICs was evaluated by digital image analysis software (Aperio membrane v9 and Aperio Genie Classifier, LEICA CAMERA AG, Wetzlar, Germany). The scoring criteria used were from a previous study (28) (TC3, ≥50%; TC2, ≥ 5 to 50%; TC1, ≥1 to < 5%; TC0, <1%; IC3, ≥10%; IC2, ≥5 to < 10%; IC1, ≥1 to < 5%; and IC0, < 1%). Additionally, all patients were divided into three groups according to tPD-L1 expression (TC0/IC0, TC1~2/IC1~2 and TC3/IC3).

### Measurement of Plasma PD-L1 mRNA

Total RNA was extracted using TRIzol Reagent (Invitrogen, Invitrogen, CA, USA), according to the manufacturer’s instructions. After the concentration and purity of the total RNA were determined, reverse transcription was performed using a PrimeScript RT Reagent Kit (TaKaRa, Dalian, China). PLACON (**Supplementary Figure 1**), a self-designed plasma external control rewarded as China patent of invention (201810102695.2), was used for amplification and comparison to detect plasma PD-L1 mRNA. The relative expression level of plasma PD-L1 mRNA in tumor patients was calculated by referring to the average expression level of plasma PD-L1 mRNA in 10 HDs samples. The formula is y=2^-(ΔCTx-ΔCT0)^. The following primer was used: PD-LI (Forward: 5’-GCTATGGTGGTGCCGACTAC-3’, Reverse: 5’-TTGGTGGTGGTGGTCTTACC-3’).

### Isolation of Exosomes From Plasma

Stored plasma samples were thawed in a water bath at 25°C. Exosomes were isolated from 200 μL of patient plasma using a Exosome Isolation Kit (Wayen Biotechnologies, Shanghai, China), according to the manufacturer’s instructions. Then, isolated exosome samples were immediately stored at -80°C until further analysis.

### Verification of Isolated Exosomes

We randomly selected one isolated exosome sample for verification. First, the size distribution of the isolated exosomes was determined through Nanosight Tracking Analysis (NTA) by utilizing ZetaView (Particle Metrix, Germany). Second, exosome morphology was analyzed by using transmission electron microscopy (TEM) (Tecnai G2 spirit BioTwin, FEI, USA). Third, exosomal proteins were subjected to SDS-PAGE followed by Western blotting (WB). The nitrocellulose membranes were blocked with 5% nonfat milk for 60 minutes at room temperature and incubated overnight at 4°C with the corresponding primary antibodies at dilutions recommended by the suppliers, followed by incubation with horseradish peroxidase (HRP)-conjugated secondary antibodies at room temperature for 1 hour. The blots were developed with enhanced chemiluminescence (ECL) PierceTM detection reagents (Thermo Scientific). CD63, CD9, and calnexin were used as exosome markers. Finally, immunoreactive proteins were visualized using a chemiluminescence detection system (FluorChem HD2, USA).

### Measurement of exoPD-L1

Exosomal PD-L1 protein was measured with a Simoa^TM^ PD-L1 Reagent Kit (Quanterix Corp, Lexington, MA). In short, all isolated exosome samples were loaded at a mass of 280 μg and then diluted with sample diluent to 130 μL for single-well detection. Standard samples were added to a 96-well plate. After the completion of the sample preparation, beads, detector, and SBG were loaded into the reagent holder, and RGP was loaded into the tube holder. Then, the sample was transferred to the Simoa Disc, using oil to seal the sample so that the signal was only in the well. Finally, pictures were taken, and the concentration was analyzed on a Simoa HD-1 platform (Quanterix Corp).

### Measurement of Soluble PD-L1

Soluble PD-L1 expression in plasma was determined using an enzyme-linked immunosorbent assay (ELISA) kit (R&D Systems, Minneapolis, USA), according to the manufacturer’s instructions. The expression level of each sample was calculated according to standard curves.

### Statistical Analysis

All experiments repeated three times, and the mean value of each sample was reported. The difference in PD-L1 mRNA and sPD-L1 expression in different subgroups was calculated by using independent-samples t-test. The difference in tPD-L1 expression and bOR in different subgroups was calculated by using Pearson’s chi-square test or Fisher’s exact test. Univariate and multivariate analyses were performed to identify independent factors of efficacy and OS. Survival analyses were performed by the Kaplan-Meier method and the log-rank test. SPSS version 23.0 (IBM, Armonk, NY, USA) was used for performing these statistical analyses. The “survival” and “survminer” packages from R software (version 3.5.2) were used for calculating the best cutoff point of each biomarker, conducting statistical calculations, and drawing Kaplan–Meier curves. A two-sided P value < 0.05 was considered statistically significant.

## Results

### Clinicopathologic Features, tPD-L1 Expression and bPD-L1 Expression in 51 patients With Various Malignancies

**Figure 1:**
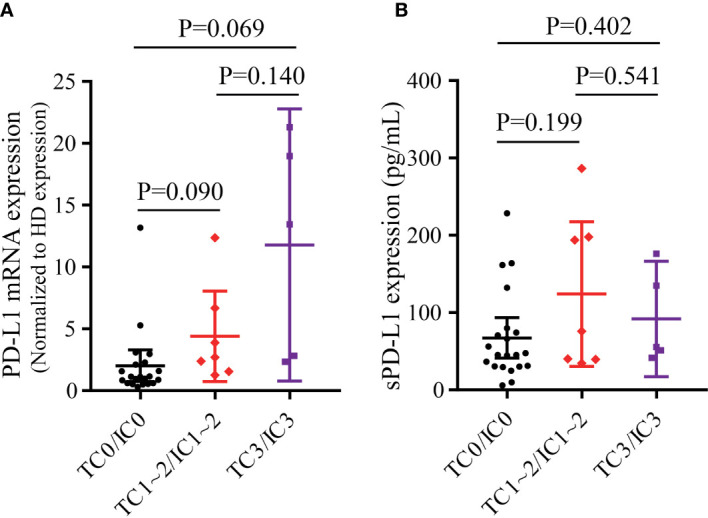
The correlation of tPD-L1 and bPD-L1 in 33 NSCLC patients. **(A)** The correlation of PD-L1 mRNA and tPD-L1. **(B)** The correlation of sPD-L1 and tPD-L1. tPD-L1, tissue PD-L1; bPD-L1, blood PD-L1; sPD-L1, soluble PD-L1; NSCLC, non-small-cell lung cancer. P values were calculated by independent-samples t-test.

Fifty-one patients with various malignancies were enrolled, including 33 NSCLC patients. Of these patients, 26 were less than 60 years old, 31 were male, 21 had a smoking history, and 33 had metastatic disease (**Table 1**). In 33 NSCLC patients, male patients had a higher PD-L1 mRNA expression than female patients. Patients with a smoking history had higher PD-L1 mRNA expression than those without a smoking history (**Supplementary Figure 2A**). No differences were found between patients younger than 60 years and older than 60 years or between patients with metastasis and without metastasis. The expression levels of tPD-L1 and sPD-L1 showed no significant differences in each subgroup (**Supplementary Figures 2B, C**). There was a trend that patients with positive tPD-L1 expression had higher PD-L1 mRNA expression (**Figure 1A**). However, the expression of sPD-L1 did not correlate with the PD-L1 mRNA expression (**Figure 1B**).

**Table 1:** Baseline clinicopathological features of 51 patients with diverse malignancies.

Clinicopathologic feature	Number of patients (%)
**Age (years)**	
<60	26 (51%)
≥60	25 (49%)
**Gender**	
Male	31 (61%)
Female	20 (39%)
**Smoking history**	
Yes	21 (41%)
No	30 (59%)
**Tumor type**	
NSCLC	33 (65%)
Others	18 (35%)
**Distant metastasis**	
Yes	33 (65%)
No	18 (35%)

NSCLC, non-small cell lung cancer.

In the overall population, the PD-L1 mRNA expression was higher in both the TC3/IC3 group (P=0.036, **Supplementary Figure 3A**) and the TC1~2/IC1~2 group (P=0.026, **Supplementary Figure 3A**) than in the TC0/IC0 group. There was also a trend that the TC3/IC3 group had a higher PD-L1 mRNA expression than the TC1~2/IC1~2 group (P=0.083, **Supplementary Figure 3A**). For sPD-L1, only the TC1~2/IC1~2 group had significantly higher expression than the TC0/IC0 group (P=0.023, **Supplementary Figure 3B**). No differences were found between the other groups. In addition, no significant differences in tPD-L1 and bPD-L1 expression were found between subgroups (**Supplementary Figure 4**).

### Dynamic Changes in bPD-L1 in 21 NSCLC Patients Treated With ICIs

**Figure 2:**
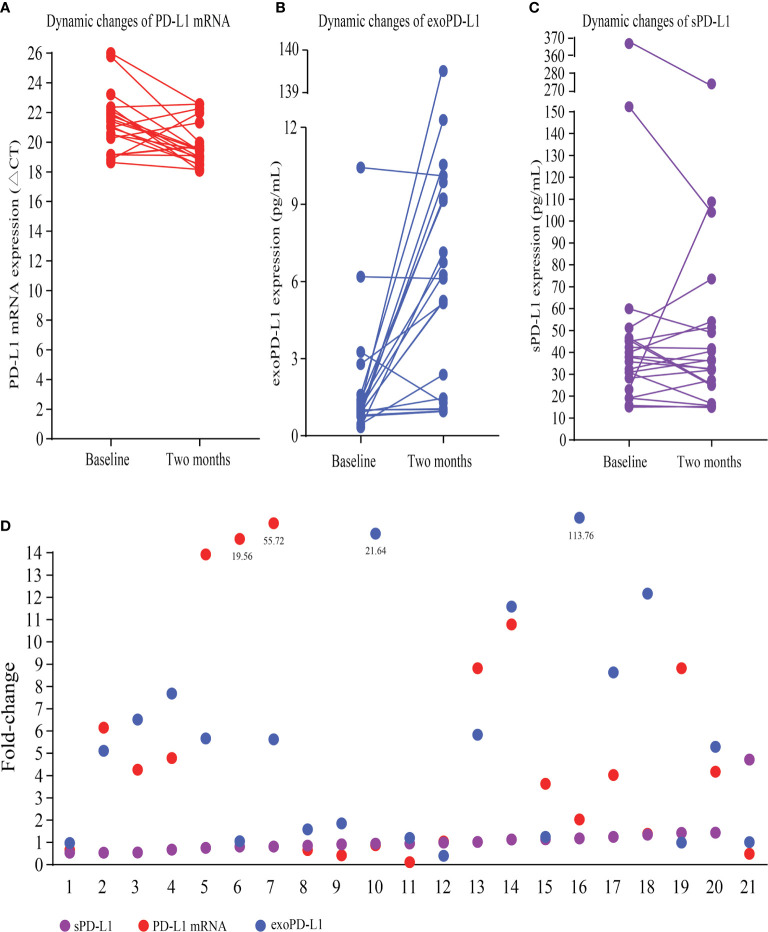
Dynamic changes in multimodal bPD-L1 expression during early treatment. **(A)** Dynamic changes in PD-L1 mRNA (CT values). **(B)** Dynamic changes in exoPD-L1. **(C)** Dynamic changes in sPD-L1. **(D)** An overview of fold changes of the three biomarkers. bPD-L1, blood PD-L1; CT, cycle threshold; exo-PD-L1, exosomal PD-L1; sPD-L1 soluble PD-L1.

Multimodal bPD-L1 expression detection, including PD-L1 mRNA, exoPD-L1, and sPD-L1, were performed in 21 advanced NSCLC patients treated with ICIs. Fifteen patients had increased PD-L1 mRNA expression at 2 months compared to baseline, while the other six patients had decreased PD-L1 mRNA expression (**Figure 2A**); the fold change ranged from 0.11 to 55.72 times. Almost all patients but three had increased exoPD-L1 expression levels (**Figure 2B**), and the fold change ranged from 0.40 to 113.76 times. For sPD-L1 expression, nine patients had increased sPD-L1 expression, while the other twelve patients had decreased sPD-L1 expression (**Figure 2C**); the fold change ranged from 0.54 to 4.72 times. An overview of the fold changes of all three kinds of bPD-L1 expression is shown in **Figure 2D**.

### Dynamic Changes in PD-L1 mRNA Expression to Predict Efficacy and OS in the Expanded 40 NSCLC Cohort

**Figure 3:**
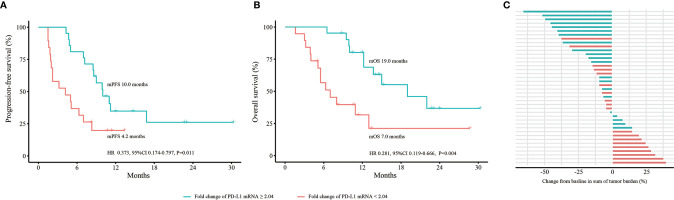
Dynamic change in PD-L1 mRNA expression to predict efficacy and OS in the expanded 40 NSCLC cohort. **(A)** PFS analysis based on fold change of PD-L1 mRNA expression. **(B)** OS analysis based on fold change of PD-L1 mRNA expression. **(C)** bOR of each patient stratified by fold change of PD-L1 mRNA expression. OS, overall survival; PFS, progression-free survival; bOR, best objective response; HR, hazard ratio; CI, confidence interval. P values were calculated by log-rank test.

To explore the role of dynamic changes in PD-L1 mRNA expression in predicting efficacy and OS, we expanded the sample size into 40 advanced NSCLC patients. According to iRECIST, 8 patients had PD; 11 had PR; 21 had SD; and no patients had CR. Blood PD-L1 mRNA expression levels at baseline and at 2 months were detected. The best cutoff value for fold change of PD-L1 mRNA expression was 2.04. The median PFS was 4.2 months (95% confidence interval [CI] 0.2-8.2 months) in patients with a fold change < 2.04. It was 10.0 months (95% CI 3.6-10.4 months) in patients with a fold change ≥ 2.04. The hazard ratio (HR) was 0.373 (fold change ≥ 2.04 vs. fold change < 2.04, 95% CI 0.174-0.797, P=0.011) (**Figure 3A**). The median OS was 7.0 months (95% CI 3.6-10.4 months) in patients with a fold change < 2.04 and 19.0 months (95% CI 9.1-28.9 months) in patients with a fold change ≥ 2.04 (HR 0.281, 95% CI 0.119-0.666, P=0.004) (**Figure 3B**). The bOR rate was 10.5% in patients with a fold change < 2.04 compared with 42.9% in patients with a fold change ≥ 2.04 (P=0.022) (**Figure 3C**).

### Dynamic Changes in exoPD-L1 and sPD-L1 to Predict Efficacy and OS in the 21 NSCLC Cohort

To verify the isolated exosomes, TEM, NTA and WB were conducted. As shown in **Supplementary Figure 5A**, the obtained exosomes had a distinctive cup shape. Then, positive marker proteins of exosomes, CD3 and CD69, were found in WB (**Supplementary Figure 5B**). A negative marker protein, calnexin, was not found (**Supplementary Figure 5B**). The size of exosomes ranged from 20 nm to 200 nm, and the average size was 117.5 nm (**Supplementary Figure 5C**).

**Figure 4:**
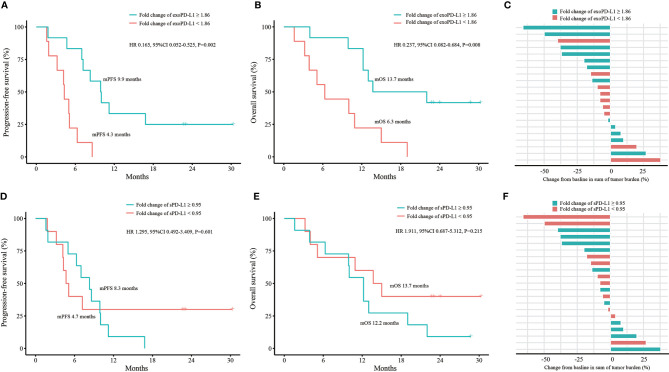
Efficacy and OS analyses based on fold change of exoPD-L1 or sPD-L1 expression in the 21 NSCLC cohort. **(A)** PFS analysis based on fold change of exoPD-L1 expression. **(B)** OS analysis based on fold change of exoPD-L1 expression. **(C)** bOR of each patient stratified by fold change of exoPD-L1 expression. **(D)** PFS analysis based on fold change of sPD-L1 expression. **(E)** OS analysis based on fold change of sPD-L1 expression. **(F)** bOR of each patient stratified by fold change of sPD-L1 expression. OS, overall survival; exoPD-L1, exosomal PD-L1; sPD-L1, soluble PD-L1; PFS, progression-free survival; bOR, best objective response; HR, hazard ratio; CI, confidence interval. P values were calculated by log-rank test.

We conducted efficacy and OS analyses according to fold changes of exoPD-L1 and sPD-L1 expression in the 21 NSCLC cohort. For exoPD-L1, patients with a fold change equals or greater than 1.86 at 2 months compared to baseline had better PFS (9.9 vs. 4.3 months, P=0.001; HR 0.165, 95% CI 0.052-0.525, P=0.002) and OS (13.7 vs. 6.3 months, P=0.004; HR 0.237, 95% CI 0.082-0.684, P=0.008) as well as a higher bOR rate (33.3% vs. 11.1%, P=0.338) (**Figures 4A–C**). For sPD-L1, no best cutoff point was found. The PFS, OS and bOR rates showed no differences (**Figures 4D–F**).

### The Combination of PD-L1 mRNA and exoPD-L1 to Predict Efficacy and OS in the 21 NSCLC Cohort

**Figure 5:**
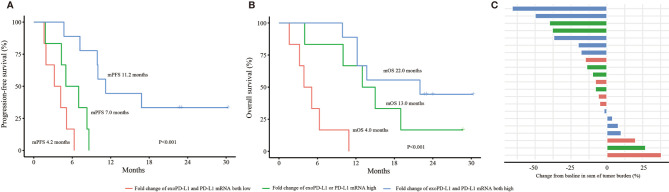
Efficacy and OS analyses based on the combination of PD-L1 mRNA and exoPD-L1 expression in the 21 NSCLC cohort. **(A)** PFS analysis based on the combination of two biomarkers. **(B)** OS analysis based on the combination of two biomarkers. **(C)** bOR of each patient stratified by the combination of two biomarkers. OS, overall survival; exoPD-L1, exosomal PD-L1; PFS, progression-free survival; bOR, best objective response. P values were calculated by log-rank test.

Univariate and multivariate analyses were performed. The results demonstrated that both the dynamic changes of PD-L1 mRNA and exoPD-L1 were independent factors for PFS and OS in the 21 NSCLC cohort (**Supplementary Tables 1** and **2**). Furthermore, we conducted survival analyses by combining the two biomarkers. Better PFS and OS were found in the combined high group compared with the single high group or the combined low group (PFS 11.2 vs. 7.0 vs. 3.2 months, P<0.001; OS 22.0 vs. 13.0 vs. 4.0 months, P<0.001) (**Figures 5A, B**). The bOR rate in the combined high group and single high group was higher than that in the combined low group (33.3% vs. 33.3% vs. 0%, P=0.269) (**Figure 5C**).

## Discussion

In the current study, we identified the correlation among tPD-L1, bPD-L1 and clinicopathologic features in 51 patients with various malignancies. Then, we explored the predictive power of multimodal bPD-L1 expression, including PD-L1 mRNA, exoPD-L1 and sPD-L1, in advanced NSCLC patients treated with ICIs.

Our results demonstrated that patients with positive tPD-L1 expression had higher PD-L1 mRNA and sPD-L1 expression in plasma, which demonstrated that bPD-L1 expression had a positive correlation with tPD-L1 expression at the same timepoints. Obviously, the acquisition of blood samples is much more convenient, less expensive, less invasive, therefore helps monitor bPD-L1 changes during ICIs treatment.

Our study first demonstrated that plasma PD-L1 mRNA could predict the efficacy and survival in NSCLC patients with ICIs treatment. The preliminary results of 21 NSCLC patients had been postered in the 2019 World Conference on Lung Cancer (29). Afterwards, we still found the same conclusion in a larger sample size of 40 patients and longer follow-up duration. Noteworthy, a report showed that a decrease of exoPD-L1 mRNA was correlated with response to ICIs treatment (30), which implied the different value of exoPD-L1 mRNA and blood PD-L1 mRNA.

Tumor-derived exosomes are extracellular vesicles with bilayer lipid membranes that carry many bioactive molecules. Tumor-derived exosomes are considered to be a key messenger in tumor progression and metastasis (31). Not surprisingly, the PD-L1 protein was found on the surface of tumor-derived exosomes (32). In vivo and *in vitro (* 33), exoPD-L1 suppressed the function of T cells by binding to PD-1. Furthermore, PD-L1-positive exosomes could spread directly from the TME to the whole body to induce systemic immunosuppression. Exosomal PD-L1 exhibits the potential to serve as a biomarker in the clinic. In a cohort of 44 melanoma patients treated with pembrolizumab (25), pretreatment exoPD-L1 expression was lower in responders than in nonresponders. In addition, pretreatment exoPD-L1 expression was positively correlated with circulating IFN-γ expression and overall tumor burden. Correspondingly, patients with an elevated exoPD-L1 expression of fold change over 2.43 had a much higher ORR. In our work, we also found an increased fold change (≥ 1.86) of exoPD-L1 in early stage of ICIs treatment indicated better efficacy and OS in NSCLC patients. In contrast, Cordonnier and colleagues (34) reported that a decrease in exoPD-L1 was associated with better response in melanoma patients. Patients with exoPD-L1 increased > 100 pg/ml had worse PFS and OS. Baseline exoPD-L1 blood levels were not associated with PFS and OS. Noticeable, the results of exoPD-L1 protein expression in this study were different from exoPD-L1 mRNA expression (30).

The source and regulation of sPD-L1 remains unclear. A paper reported that sPD-L1 might be derived from TCs and retained the PD-1-binding domain (35). Plasma sPD-L1 could systemically impair host immunity and promote tumor progression. Zhou et al. (26) reported that higher initial sPD-L1 expression was prone to disease progression in malignant melanoma patients with ICIs treatment, while over 1.5-fold increase of sPD-L1 expression at five months showed a positive correlation with PR. Okuma et al. (36) reported that a higher baseline sPD-L1 expression was negatively associated with OS and ORR in NSCLC patients receiving nivolumab. Costantini et al. (37) demonstrated that high sPD-L1 at 2 months and increase of sPD-L1 concentrations were associated with poor response and absence of clinical benefit in NSCLC patients treated by nivolumab. In the current study, the sPD-L1 change showed no correlation with efficacy and OS, which were different from the previous studies.

Additionally, tPD-L1 expression in the TME increased at early stage of treatment in patients who responded to ICIs (14, 15). These data suggested that in the early stage of ICIs treatment, both tPD-L1 and bPD-L1 expression could be upregulated. The underlying mechanism on higher level of PD-L1 on TCs could be a feedback and T-cell reinvigoration of immune response. Nevertheless, elevated PD-L1 expression couldn’t play its role of negative immune regulation because ICIs therapy had blocked the interaction of PD-1 and PD-L1.

Furthermore, our work demonstrated that the combination of blood PD-L1 mRNA and exoPD-L1 could better determine NSCLC subgroups who may benefit from ICIs treatment. Though patients might have a fold change of exoPD-L1 < 1.86, part of them could have a fold-change of PD-L1 mRNA ≥ 2.04. These patients had better efficacy and OS than those with fold changes of PD-L1 mRNA and exoPD-L1 both low. In addition, patients with both a fold change of exoPD-L1 ≥ 1.86 and a fold change of PD-L1 mRNA ≥ 2.04 had the best efficacy and OS outcomes.

Besides the above indexes, bPD-L1 was also found on the surface of circulating tumor cells (CTCs) (38, 39). Nicolazzo et al. (40) monitored PD-L1 expression on CTCs from baseline to 6 months in 24 advanced NSCLC patients treated with nivolumab. The results showed that those with continuous PD-L1 expression experienced disease progression, while those with negative PD-L1 expression at 6 months obtained tumor response. Another work (41) got the same results. However, Yue et al. (42) reported the opposite conclusion that that patients with a higher PD-L1^high^ CTCs (abundance over 20%) at baseline had an obvious disease control and longer PFS, and decreased PD-L1^high^ CTCs at 9 weeks were associated with disease control. More research in this field is necessary.

To the best of our knowledge, this is the first report of changes in PD-L1 mRNA and exoPD-L1 to predict the efficacy of ICIs treatment. Dynamic liquid biopsy of multimodal PD-L1 is a good way to occasionally monitor patients during ICIs treatment. Our findings have crucial clinical significance. First, we know which patients would benefit from ICIs treatment and which subgroups would not. Second, we may pay more attention to the potential disease progression in those patients who have lower fold changes in exoPD-L1 and PD-L1 mRNA during early treatment. Some salvage therapy, such as chemotherapy, radiotherapy, or antivascular drug, could be intervened earlier than imaging progress. Third, we built a patent product of external control for blood mRNA detection to make the blood PD-L1 mRNA a standard biomarker to evaluate the clinical benefit of ICIs treatment.

There are some limitations in our work. The sample size is relatively small. In the future, we plan to design a prospective clinical trial to confirm the value of blood PD-L1 biomarker from ICIs treatment in NSCLC patients. We did not recruit early-stage NSCLC patients. Thus, we do not know if bPD-L1 is an efficacy biomarker for neoadjuvant ICIs treatment before surgery, or adjuvant ICIs treatment after surgery. All these questions could be explored and solved in future studies.

## Conclusions

In summary, bPD-L1 expression has a positive correlation with tPD-L1 expression in various malignancies. Upregulated expression of blood PD-L1 mRNA and exoPD-L1 predicted good efficacy and survival for ICIs treatment. In particular, the combination of these two biomarkers could screen better subpopulation. Our viewpoint of dynamic changes of blood PD-L1 mRNA and exoPD-L1 could serve as novel biomarkers in NSCLC patients with ICIs treatment.

## Data Availability Statement

The raw data supporting the conclusions of this article will be made available by the authors, without undue reservation.

## Ethics Statement

The studies involving human participants were reviewed and approved by the ethics committee of the Xinqiao Hospital of Army Medical University (2016-No.054-01, 2017-No.011-01). The patients/participants provided their written informed consent to participate in this study.

## Author Contributions

Contributions to the conception: LuZ, JS. Design of the work: ZC, WZ. The acquisition, analysis, and interpretation of data: KN, XZ, LiZ, ZX, YY. The creation of new software used in the work: FL, LM. Draft the work and substantively revised it: QY, MC, JG, LuZ, JS. All authors contributed to the article and approved the submitted version. All authors have agreed both to be personally accountable for the author’s own contributions and to ensure that questions related to the accuracy or integrity of any part of the work, even ones in which the author was not personally involved, are appropriately investigated, resolved, and the resolution documented in the literature.

## Conflict of Interest

The authors declare that the research was conducted in the absence of any commercial or financial relationships that could be construed as a potential conflict of interest.

## Supplementary Material

Supplementary Figure 1 PLACON sequence. We selected a conserved sequence from the genome of *Caenorhabditis elegans*, which we named PLACON. The following primers were used: (Forward: 5’-AGTGCAGGGTCCGAGGTATT-3’, Reverse: 5’-CGACTCTACAACGACCGTGA-3’). The PLACON sequence: 5’-CUCGCUAACGACUCUACAACGACCGUGAAUUCAAGCGCCGCUUGGAUGUCCGC-3’ **(A)**. Then, we identified that PLACON had good specificity through BLAST (https://www.ncbi.nlm.nih.gov/). No cross-correlation with the human genome was found. Then, we identified the amplification ability of PLACON by comparing it with internal references, including GAPDH and β-actin, in plasma from 8 patients with malignant tumors and cancer cell supernatants. As shown in **(B, C)** the CT value of PLACON was much lower than that of GAPDH and β-actin. In conclusion, PLACON is specific, and it has an obvious amplification advantage. It could be used as an external reference for the quantitative detection of mRNA in plasma. CT, cycle threshold.

Supplementary Figure 2 The differences of tPD-L1 and bPD-L1 between subgroups in 33 NSCLC patients. **(A)** Comparison of PD-L1 mRNA expression between subgroups. **(B)** Comparison of sPD-L1 expression between subgroups. **(C)** Comparison of tPD-L1 expression between subgroups. tPD-L1, tissue PD-L1; bPD-L1, blood PD-L1; NSCLC, non-small cell lung cancer; sPD-L1, soluble PD-L1. P values were calculated by independent-samples t-test **(A, B)** and Pearson’s chi-square test or Fisher’s exact test **(C)**.

Supplementary Figure 3 The correlation of tPD-L1 and bPD-L1 in 51 patients with various malignancies. **(A)** The correlation of PD-L1 mRNA and tPD-L1. **(B)** The correlation of sPD-L1 and tPD-L1. tPD-L1, tissue PD-L1; bPD-L1, blood PD-L1; sPD-L1, soluble PD-L1; NSCLC, non-small-cell lung cancer. P values were calculated by independent-samples t-test.

Supplementary Figure 4 The differences of tPD-L1 and bPD-L1 between subgroups in 51 patients with various malignancies. **(A)** Comparison of PD-L1 mRNA expression between subgroups. **(B)** Comparison of sPD-L1 expression between subgroups. **(C)** Comparison of tPD-L1 expression between subgroups. tPD-L1, tissue PD-L1; bPD-L1, blood PD-L1; sPD-L1, soluble PD-L1. P values were calculated by independent-samples t-test **(A, B)** and Pearson’s chi-square test or Fisher’s exact test **(C)**.

Supplementary Figure 5 Verification of exosomes. **(A)** Exosome morphology detected by TEM. **(B)** Positive markers (CD9, CD63) and a negative marker (calnexin) of exosomes detected by WB. **(C)** Size analysis of exosomes through NTA. TEM, transmission electron microscopy; WB, western blotting; NTA, nanosight tracking analysis.
