# Supplementary figures and images for: Novel Biomarkers of Dynamic Blood PD-L1 Expression for Immune Checkpoint Inhibitors in Advanced Non-Small-Cell Lung Cancer Patients
Source: Front Immunol. 2021 Apr 16;12:665133. doi: 10.3389/fimmu.2021.665133 (PMC8085403; doi:10.3389/fimmu.2021.665133)

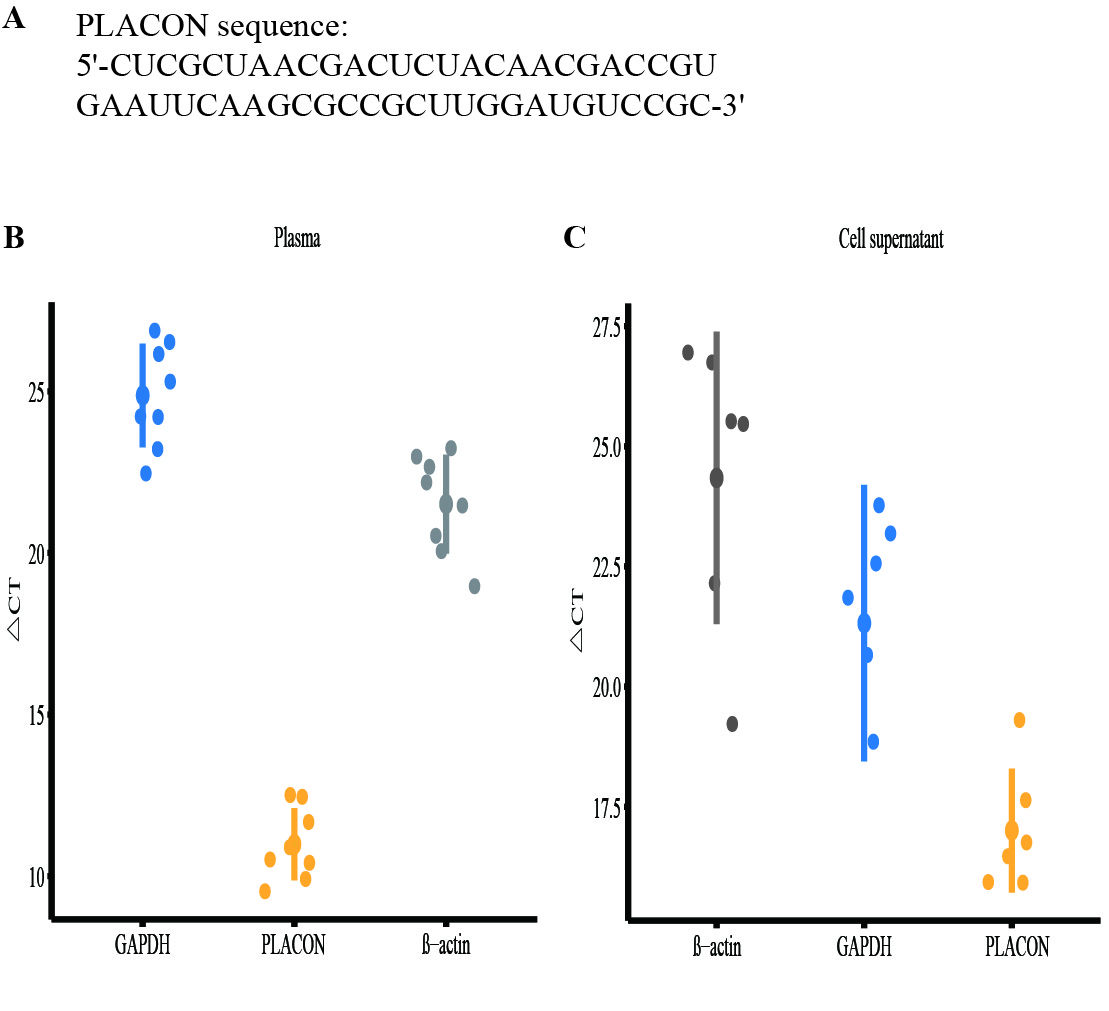

Supplement: Supplementary Figure 1 — PLACON sequence. We selected a conserved sequence from the genome of Caenorhabditis elegans, which we named PLACON. The following primers were used: (Forward: 5’-AGTGCAGGGTCCGAGGTATT-3’, Reverse: 5’-CGACTCTACAACGACCGTGA-3’). The PLACON sequence: 5’-CUCGCUAACGACUCUACAACGACCGUGAAUUCAAGCGCCGCUUGGAUGUCCGC-3’ (A). Then, we identified that PLACON had good specificity through BLAST (https://www.ncbi.nlm.nih.gov/). No cross-correlation with the human genome was found. Then, we identified the amplification ability of PLACON by comparing it with internal references, including GAPDH and β-actin, in plasma from 8 patients with malignant tumors and cancer cell supernatants. As shown in (B, C) the CT value of PLACON was much lower than that of GAPDH and β-actin. In conclusion, PLACON is specific, and it has an obvious amplification advantage. It could be used as an external reference for the quantitative detection of mRNA in plasma. CT, cycle threshold. [file Image_1.jpeg]

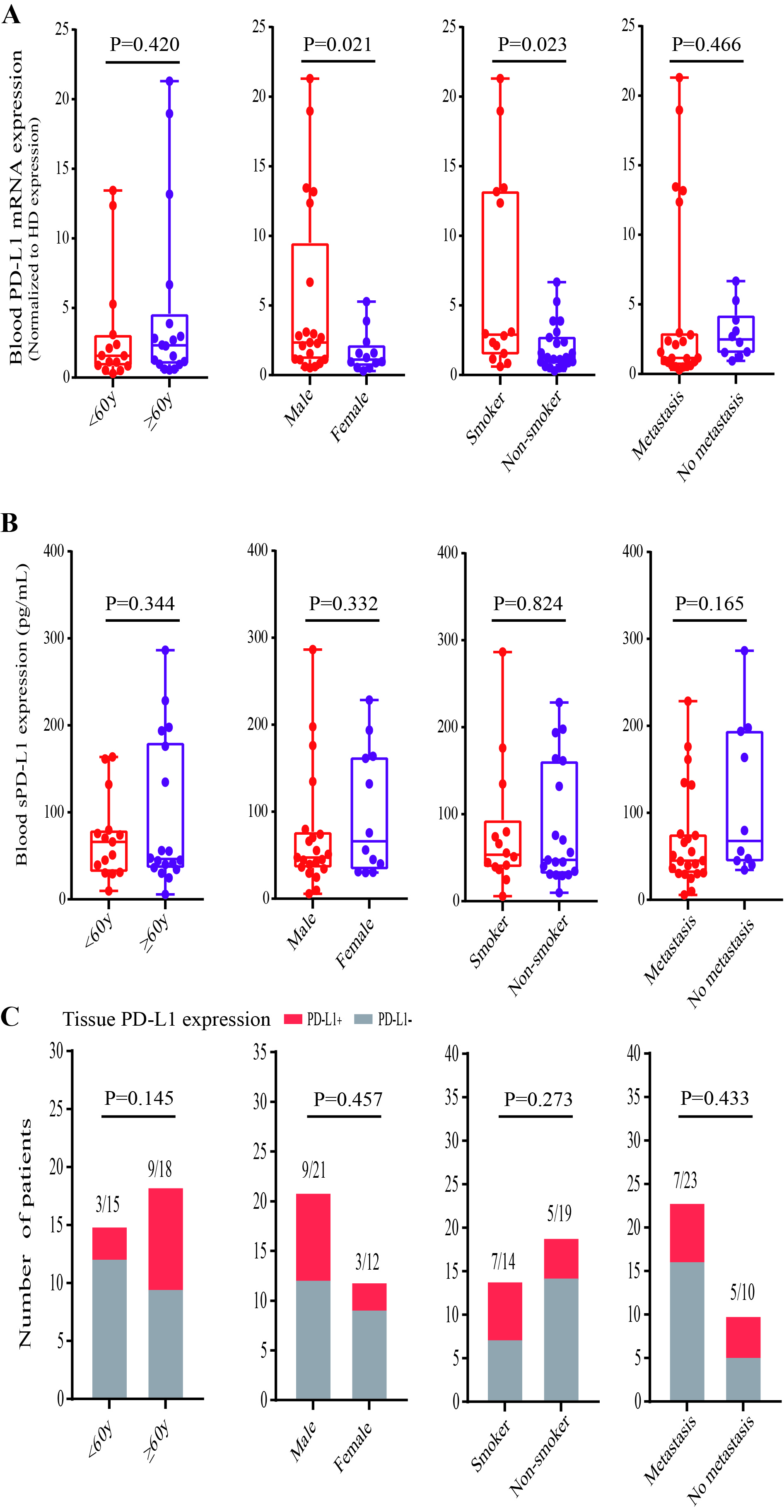

Supplement: Supplementary Figure 2 — The differences of tPD-L1 and bPD-L1 between subgroups in 33 NSCLC patients. (A) Comparison of PD-L1 mRNA expression between subgroups. (B) Comparison of sPD-L1 expression between subgroups. (C) Comparison of tPD-L1 expression between subgroups. tPD-L1, tissue PD-L1; bPD-L1, blood PD-L1; NSCLC, non-small cell lung cancer; sPD-L1, soluble PD-L1. P values were calculated by independent-samples t-test (A, B) and Pearson’s chi-square test or Fisher’s exact test (C). [file Image_2.jpeg]

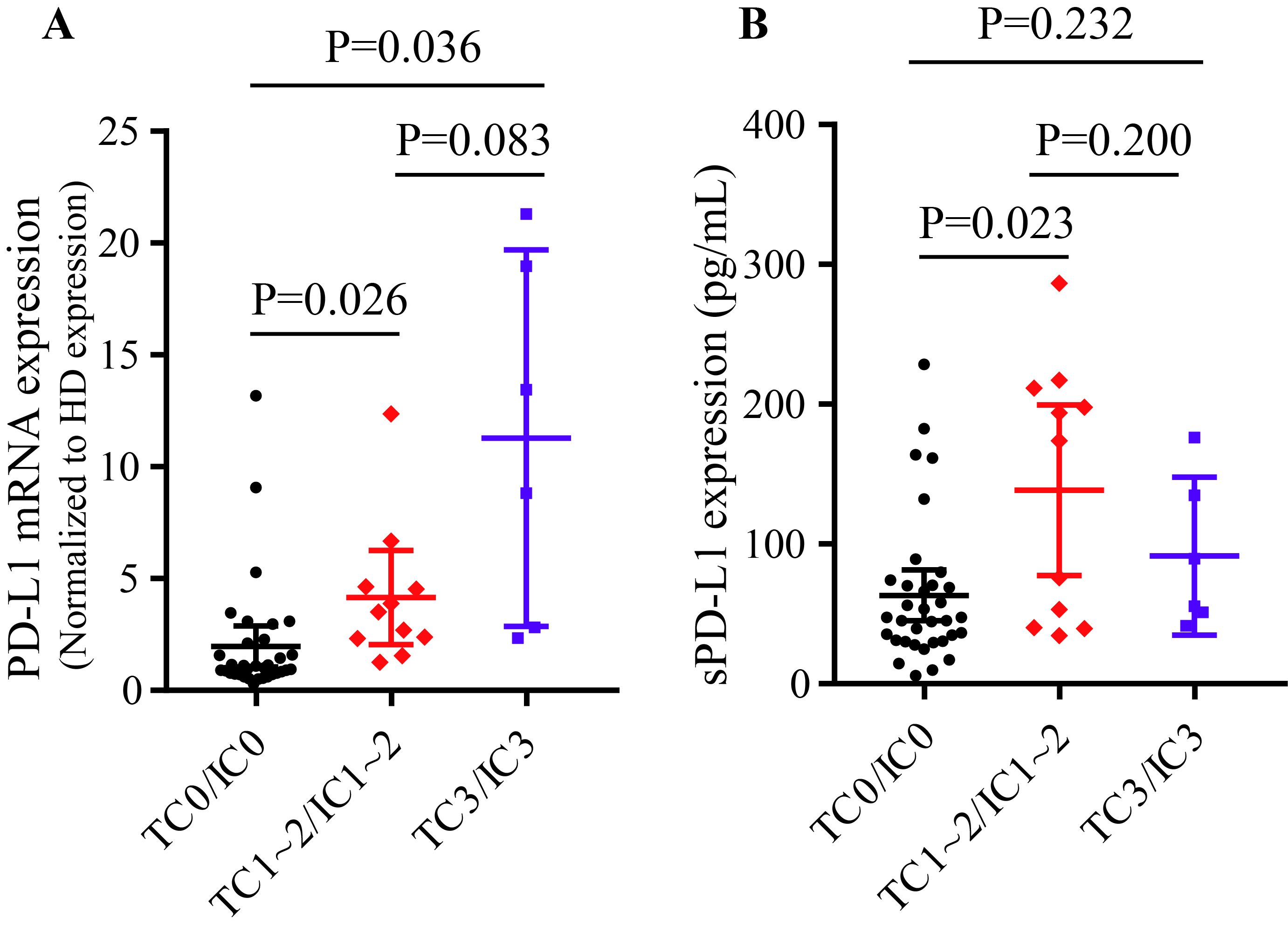

Supplement: Supplementary Figure 3 — The correlation of tPD-L1 and bPD-L1 in 51 patients with various malignancies. (A) The correlation of PD-L1 mRNA and tPD-L1. (B) The correlation of sPD-L1 and tPD-L1. tPD-L1, tissue PD-L1; bPD-L1, blood PD-L1; sPD-L1, soluble PD-L1; NSCLC, non-small-cell lung cancer. P values were calculated by independent-samples t-test. [file Image_3.jpeg]

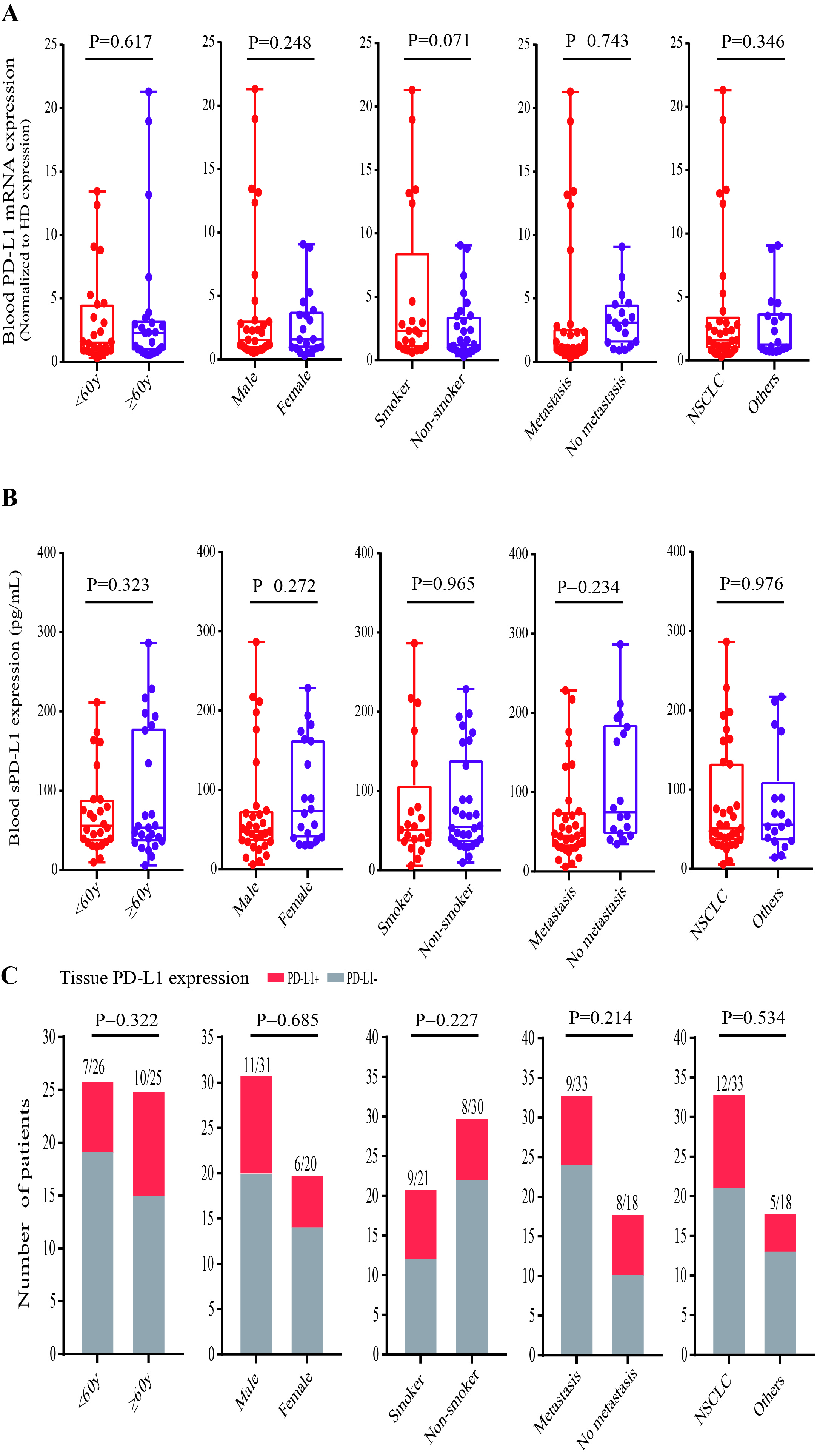

Supplement: Supplementary Figure 4 — The differences of tPD-L1 and bPD-L1 between subgroups in 51 patients with various malignancies. (A) Comparison of PD-L1 mRNA expression between subgroups. (B) Comparison of sPD-L1 expression between subgroups. (C) Comparison of tPD-L1 expression between subgroups. tPD-L1, tissue PD-L1; bPD-L1, blood PD-L1; sPD-L1, soluble PD-L1. P values were calculated by independent-samples t-test (A, B) and Pearson’s chi-square test or Fisher’s exact test (C). [file Image_4.jpeg]

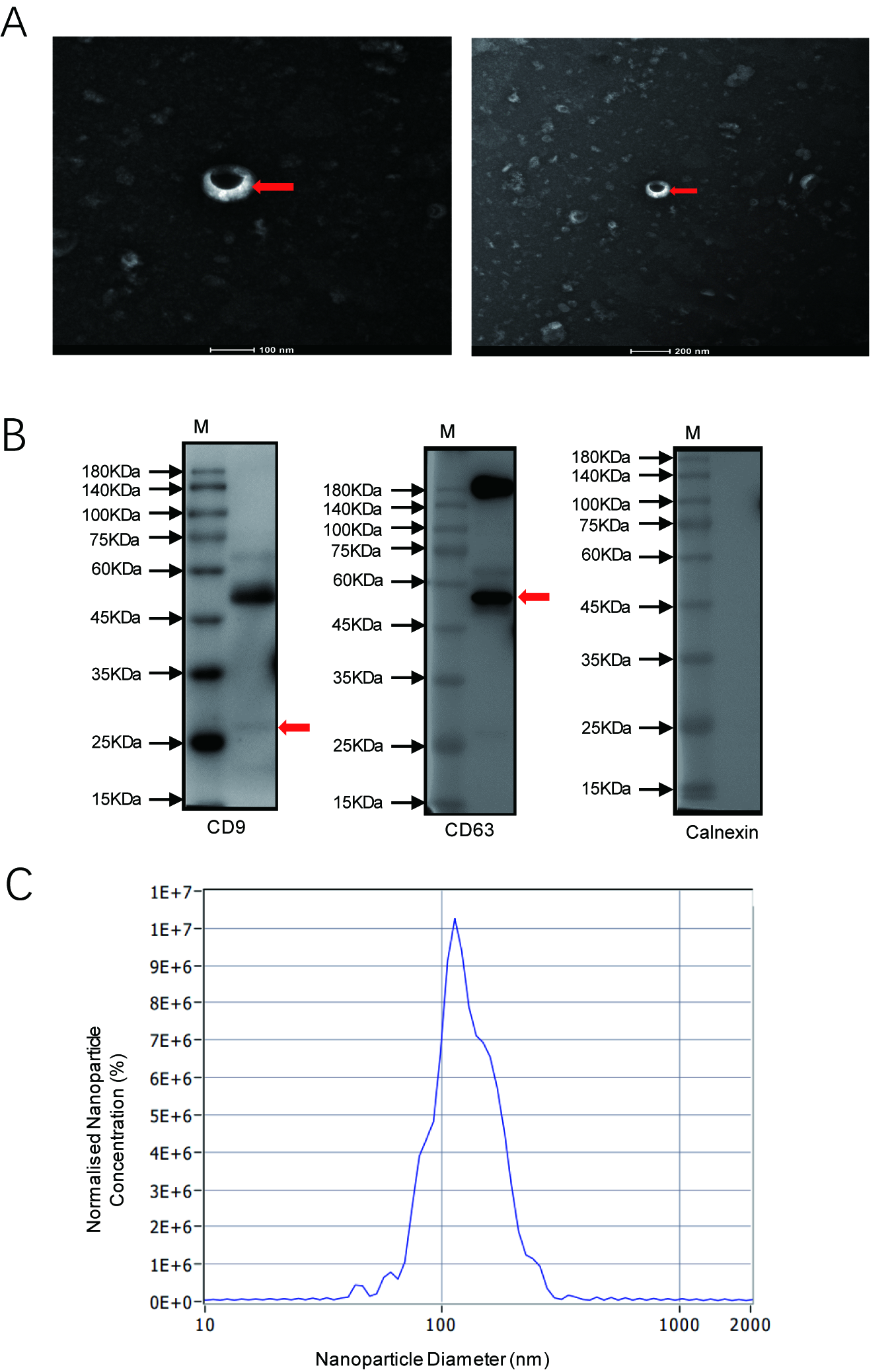

Supplement: Supplementary Figure 5 — Verification of exosomes. (A) Exosome morphology detected by TEM. (B) Positive markers (CD9, CD63) and a negative marker (calnexin) of exosomes detected by WB. (C) Size analysis of exosomes through NTA. TEM, transmission electron microscopy; WB, western blotting; NTA, nanosight tracking analysis. [file Image_5.tif]
