# Supplementary material for: Novel Biomarkers of Dynamic Blood PD-L1 Expression for Immune Checkpoint Inhibitors in Advanced Non-Small-Cell Lung Cancer Patients
Source: Front Immunol. 2021 Apr 16;12:665133. doi: 10.3389/fimmu.2021.665133 (PMC8085403; doi:10.3389/fimmu.2021.665133)
Supplement: Supplementary file 6 [file Table_1.docx]

Supplementary Table 1: Univariate and multivariate analyses of the effect of the dynamic change of PD-L1 mRNA and exosomal PD-L1 on progression-free survival

| **Variable** | **Univariate** | | **Multivariate** | |
| --- | --- | --- | --- | --- |
|  | **HR (95% CI)** | **P-Value** | **HR (95% CI)** | **P-Value** |
| **Fold change of PD-L1 mRNA** |  |  |  |  |
| < 2.04 | Reference | — | Reference | — |
| ≥ 2.04 | 0.149 (0.044-0.507) | 0.002 | 0.197 (0.053-0.729) | 0.015 |
| **Fold change of exosomal PD-L1** |  |  |  |  |
| < 1.86 | Reference | — | Reference | — |
| ≥ 1.86 | 0.165 (0.052-0.525) | 0.002 | 0.216 (0.061-0.767) | 0.018 |
| HR, hazard ratio; CI, confidence interval. | | | | |
