# Supplementary material for: Novel Biomarkers of Dynamic Blood PD-L1 Expression for Immune Checkpoint Inhibitors in Advanced Non-Small-Cell Lung Cancer Patients
Source: Front Immunol. 2021 Apr 16;12:665133. doi: 10.3389/fimmu.2021.665133 (PMC8085403; doi:10.3389/fimmu.2021.665133)
Supplement: Supplementary file 7 [file Table_2.docx]

Supplementary Table 2: Univariate and multivariate analyses of the effect of the dynamic change of PD-L1 mRNA and exosomal PD-L1 on overall survival

| **Variable** | **Univariate** | | **Multivariate** | |
| --- | --- | --- | --- | --- |
|  | **HR (95% CI)** | **P-Value** | **HR (95% CI)** | **P-Value** |
| **Fold change of PD-L1 mRNA** |  |  |  |  |
| < 2.04 | Reference | — | Reference | — |
| ≥ 2.04 | 0.291 (0.106-0.800) | 0.017 | 0.289 (0.098-0.855) | 0.025 |
| **Fold change of exosomal PD-L1** |  |  |  |  |
| < 1.86 | Reference | — | Reference | — |
| ≥ 1.86 | 0.239 (0.083-0.688) | 0.008 | 0.238 (0.078-0.721) | 0.011 |
| HR, hazard ratio; CI, confidence interval. | | | | |
